# Glucose Transporter 1 and Monocarboxylate Transporters 1, 2, and 4 Localization within the Glial Cells of Shark Blood-Brain-Barriers

**DOI:** 10.1371/journal.pone.0032409

**Published:** 2012-02-28

**Authors:** Carolina Balmaceda-Aguilera, Christian Cortés-Campos, Manuel Cifuentes, Bruno Peruzzo, Lauren Mack, Juan Carlos Tapia, Karina Oyarce, María Angeles García, Francisco Nualart

**Affiliations:** 1 Laboratory of Neurobiology and Stem Cells, Department of Cellular Biology, University of Concepcion, Concepción, Chile; 2 Laboratory of Cellular Biology, Department of Cellular Biology, University of Concepcion, Concepción, Chile; 3 Department of Cellular Biology, Genetics and Physiology, Faculty of Sciences, Malaga University, Málaga, Spain; 4 Anatomy, Histology and Pathology Institute, Faculty of Medicine, Universidad Austral de Chile, Valdivia, Chile; 5 Departments of Biochemistry and Molecular Biophysics and Neuroscience, Columbia University, New York, New York, United States of America; University of Chicago, United States of America

## Abstract

Although previous studies showed that glucose is used to support the metabolic activity of the cartilaginous fish brain, the distribution and expression levels of glucose transporter (GLUT) isoforms remained undetermined. Optic/ultrastructural immunohistochemistry approaches were used to determine the expression of GLUT1 in the glial blood-brain barrier (gBBB). GLUT1 was observed solely in glial cells; it was primarily located in end-feet processes of the gBBB. Western blot analysis showed a protein with a molecular mass of 50 kDa, and partial sequencing confirmed GLUT1 identity. Similar approaches were used to demonstrate increased GLUT1 polarization to both apical and basolateral membranes in choroid plexus epithelial cells. To explore monocarboxylate transporter (MCT) involvement in shark brain metabolism, the expression of MCTs was analyzed. MCT1, 2 and 4 were expressed in endothelial cells; however, only MCT1 and MCT4 were present in glial cells. In neurons, MCT2 was localized at the cell membrane whereas MCT1 was detected within mitochondria. Previous studies demonstrated that hypoxia modified GLUT and MCT expression in mammalian brain cells, which was mediated by the transcription factor, hypoxia inducible factor-1. Similarly, we observed that hypoxia modified MCT1 cellular distribution and MCT4 expression in shark telencephalic area and brain stem, confirming the role of these transporters in hypoxia adaptation. Finally, using three-dimensional ultrastructural microscopy, the interaction between glial end-feet and leaky blood vessels of shark brain was assessed in the present study. These data suggested that the brains of shark may take up glucose from blood using a different mechanism than that used by mammalian brains, which may induce astrocyte-neuron lactate shuttling and metabolic coupling as observed in mammalian brain. Our data suggested that the structural conditions and expression patterns of GLUT1, MCT1, MCT2 and MCT4 in shark brain may establish the molecular foundation of metabolic coupling between glia and neurons.

## Introduction

Based on functional studies in a limited number of sub-mammalian vertebrates (e.g., primarily jawless vertebrates, cartilaginous fishes, and amphibians), it appears that the physiological characteristics of the blood-brain-barriers (BBBs) are similar among the vertebrate classes [1]. However, comparative ultrastructural analyses of the BBB of cartilaginous fishes, including sharks and skates, demonstrated leaky blood vessels surrounded by a sheath of glial foot processes, which contain tight junctions that prevent the diffusion of various molecules [1], [2], [3], [4], [5]. Therefore, it is postulated that if the glial cells (i.e., astrocytes) within the BBB of cartilaginous fish participate in the transcellular transport of glucose, they may overexpress molecules involved in carrier-mediated transport mechanisms [6] and metabolic coupling such as glucose transporters (GLUT) and monocarboxylate transporters (MCT) [7], [8], [9], [10]

In mammals and birds, several GLUT isoforms have been molecularly identified [11], [12], [13], [14], [15], [16], [17]. GLUT1 is highly expressed in mammalian endothelial cells that form the BBB and in epithelial cells of the choroid plexus, which form the cerebrospinal fluid (CSF) -blood barrier, thereby contributing greatly to the efficient acquisition of glucose by the brain [18], [19]. In contrast, GLUT1 is expressed to a much lesser degree in neurons and astrocytes of the mammalian brain; rather GLUT3, a high affinity transporter, is expressed in neurons [20], [21], [22], [23].

The general expression and localization of GLUTs has been primarily studied in mammalian species; a detailed analysis in submammalian vertebrates has yet to be performed. In teleost fish, the existences of at least two proteins that have high homology with mammalian GLUT1 and GLUT4 have been identified [24], [25]. Although glucose is vital for the metabolic activity of the cartilaginous and bony fishes brain, no immunohistochemical data analyzing the distribution and expression levels of GLUT isoforms involved in the acquisition of glucose exists. However, glucose permeability analysis of the BBB in shark (*Squalus acanthias*) indicated that the transport of 3-O-methyl-glucose is mediated by a saturable and stereospecific component, suggesting the expression of a transporter similar to GLUT1 [6].

The MCTs are a family of proteins, which contain 14 isoforms. MCT1-MCT4 transport lactate, pyruvate and ketone bodies [26], [27]. Isoforms MCT1, MCT2, MCT3, MCT4, MCT6, MCT7, MCT8, MCT11 and MCT14 have been detected in mammalian brain [26], [27], [28], [29], [30], [31], [32]. MCT1 has been primarily been found in the endothelial cells of capillaries and astrocytes [29], [30], [32], [33], [34], [35] while MCT2 is a neuronal transporter [36]. Ultrastructural studies have demonstrated that MCT2 is localized in the dendrites of neurons [35], [37]. MCT4 is found in astroglial cells, specifically in Bergmann glia of the cerebellum and astrocytes of various areas, including the hippocampus [35] and in rat hypothalamic tanycytes [33]. In species other than mammals, almost no information concerning brain MCT localization exists. The presence of the BBB in the processes of glial cells may promote a metabolic coupling mechanism in cartilaginous fish; glucose may directly enter into the glia cell, thereby generating lactate, which may subsequently be used by neurons. Because the metabolic process may be an effective mechanism for obtaining glucose in the brain, it is important to study the expression and distribution of GLUTs and MCTs.

Previous studies have demonstrated that hypoxia modifies the expression and distribution of MCTs and GLUTs [38], [39], [40]. In addition, expression of GLUT-1 and -3 in mammals is induced by hypoxic stress and presumably mediated by the transcription factor, hypoxia inducible factor-1 (HIF-1), via binding to hypoxia-responsive DNA elements within the promoters [23], [41]. In mammals, the MCT4 gene promoter contains elements responsive to hypoxia, which is similar to that described for HIF-1α [39], [42]. Similarly, in the gills of the teleost fish *Danio rerio* (zebrafish) [38], changes in MCT4 mRNA expression levels have been reported in response to hypoxia. Therefore, changes in the expression and distribution of GLUT and MCT may represent a pathophysiological condition used by the shark brain for responding to hypoxia.

In the present study, various experimental methods were used to analyze the expression and distribution of GLUT1, MCT1, MCT2, and MCT4 in normal and hypoxic shark brain. Their distribution and modulation in response to hypoxic conditions, may suggest a glia-neuronal metabolic coupling in shark brain.

## Results

### Glia cell distribution and end-feet processes in shark brain

**Figure 1:**
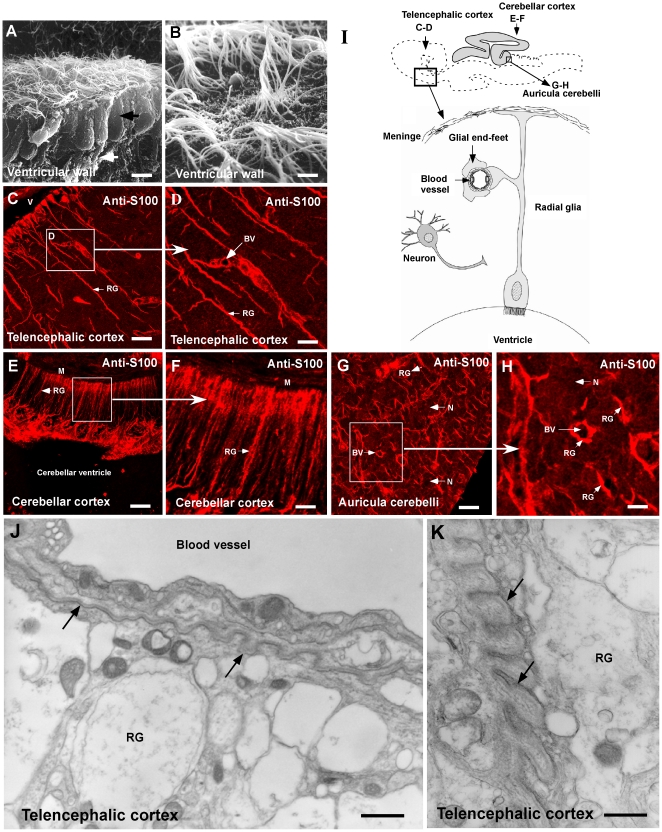
Cellular characteristics and distribution of glial cells in the shark brain. A–B, Scanning electron microscopy of glial cells contacting the CSF (arrow). C–H, Shark (*S. chilensis*) sagittal brain sections using anti-S100 antibody in the telencephalic area (C–D), cerebellar cortex (E–F) and auricula cerebelli (G–H). I, Schematic representation of radial glial cells, neurons and endothelial cells present in the shark brain. Glial end-feet are in contact with blood vessel endothelial cells. J–K, Ultrastructural analysis of blood vessels and radial glia end-feet. The basal membrane of the blood vessel is shown with arrows. BV: blood vessel, N: neuron, RG: radial glia processes, V: ventricle. Scale bar: A and B, 10 µm; C–H, 20 αm; J–K, 2 αm.

Several antibodies specific for S100a, vimentin, GFAP, GLAST, and 3CB2 were used to identify radial glia and astrocytes in *S. chilensis*. A positive reaction was seen only with anti-S100a (Fig. 1) and anti-3CB2 antibodies (data not shown). Specifically, in the telecenphalic cortex, the ciliated glial cells that cover the ventricle wall (Fig. 1A–B) present an intense immunoreaction for anti-S100 (Fig. 1C–D). The processes of these cells cross the brain neuropile, and their branches surround blood vessels (Fig. 1C–D, BV). Furthermore, S100-positive glial cells cross the brain neuropile and contact the meninges (data not shown). In the cerebellar cortex, auricula cerebelli (Fig. 1E–F and G–H), mesencephalic tectum and brain stem, the distribution of the radial glia was similar to that observed in the telecephalon.

The relationship between the processes and branches of the radial glia and blood vessels was further studied by ultrastructural analysis; glial cells of shark brain possessed a distribution similar to radial glia in mammalian brain (Fig. 1I). The processes of the radial glia with electron-lucent expansion (similar to astrocyte end-feet) contact the basal membrane of blood vessels (Fig. 1J–K). Most of the blood vessels have perivascular spaces with folded basal membranes (Fig. 1J–K, arrows).

**Figure 2:**
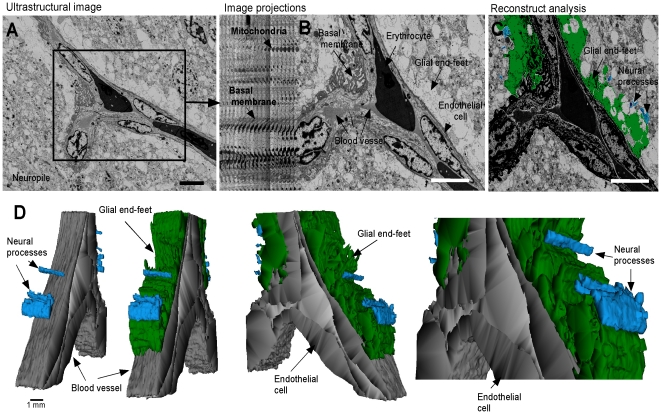
Three-dimensional reconstruction of glial end-feet in the brain cortex. A, Ultrastructural images from shark brain (*S. chilensis*) using low magnification. The telencephalic neurophil showed neural processes and glial cell end-feet contacting the blood vessel. B–C, Forty ultrathin sections (50 nm) were used to create a three-dimensional reconstruction of dendritic (blue) and glial end-feet (green) around a blood vessel (gray). D, Most of the blood vessel is surrounded by glial end-feet that form an irregular barrier. Scale bar: A–C, 15 µm.

Ultrastructural microscopy was also employed to analyze the shark brain neuropile (*S. chilensis*) with low magnification and reconstruct the glial end-feet processes by analyzing forty ultrathin sections (Fig. 2A–B). Three-dimensional reconstruction of dendritic (blue) and glial end-feet (green) around the blood vessel (gray) (Fig. 2C) demonstrated that the blood vessels are surrounded by glial end-feet that form an irregular but continuous BBB between the neuropile and endothelial cells (Fig. 2D).

### GLUT1 distribution in shark brain

**Figure 3:**
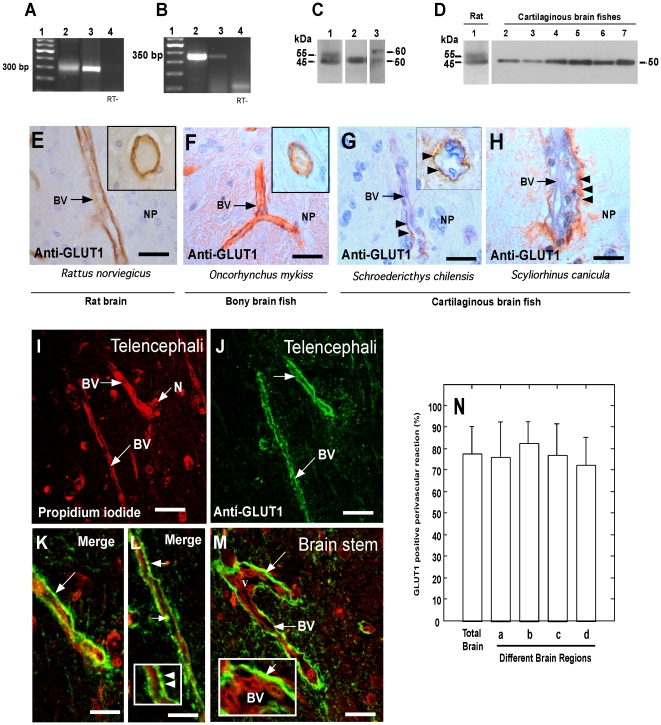
GLUT1 expression and localization in shark brain. A–B, RT-PCR analysis of GLUT1 expression using primer sets 1 (A) and 2 (B) and total RNA isolated from the following tissues and treatments: Lane 2, shark brain *(S. chilensis)*; Lane 3, rat brain; Lane 4, shark brain (-RT). Lane 1 contains the DNA ladder. C–D, Western blot analysis of GLUT1. C, Total protein extracts were prepared from rat brain (lane 1), shark brain (*S. chilensis*, lane 2) and bony fish brain (lane 3). D, Total protein extracts were prepared from rat brain (lane 1) and the following regions of the shark brain (*S. chilensis*): total brain (lane 2), telencephalic cortex (lane 3), diencephalon (lane 4), mesencephalic tectum (lane 5), cerebellar cortex (lane 6), and brain stem (lane 7). E–H, Immunohistochemistry analysis of GLUT1 expression in the telencephalic area. GLUT1 is localized in the endothelial cells of rat and bony fish brain (E, F). The insets show a cross-section of the blood vessels. In shark brain from *S. chilensis* and *S. canicula* (G, H), GLUT1 is expressed in the perivascular zone (arrows). The inset (G) shows a cross -section of the blood vessel with a positive reaction in the perivascular region (arrows). I–M, Immunofluorescence and confocal microscopy using anti-GLUT1 antibodies in the telencephalic area of *S. chilensis*. Tissue was also stained with propidium iodide to identify cells of the brain and the endothelial cells of the blood vessels (I). GLUT1 is localized in perivascular structures (arrows; K–M), with little co-localization with propidium iodide (inset in L and M). N, Percentage of number of vessels in *S. chilensis* brain with perivascular GLUT1 reactivity. Data represent the means ± SD from four independent experiments, telencephalic cortex (a), diencephalon (b), mesencephalic area (c) and cerebellum (d). BV: blood vessel, N: neuron, NP: neuropile. Scale bar: E–H, 30 µm; I–J, 50 µm; K–M, 20 µm.

Two different primer sets were used to demonstrate by RT-PCR the expression of GLUT1 mRNA in the brain of *S. chilensis* (Fig. 3A, B). The first set of primers was designed for the EST sequence of GLUT1 in *Squalus acanthias* (CX197025), which amplifies a 281 bp fragment (Fig. 3A, lane 3) similar to the fragment obtained using mRNA isolated from rat brain (Fig. 3A, lane 2). The sequence of this fragment (data not shown) contains a 74% identity with rat GLUT1 mRNA. The second set of primers generated a 350 bp fragment (Fig. 3B, lane 2), whose sequence presented 80% identity with rat GLUT1 mRNA. Both fragments generated a theoretical sequence of 127 amino acids, which contains 76% identity with the GLUT1 sequence for rat.

To determinate GLUT1 expression in rat, bony fish and shark brains, polyclonal antibodies and Western blot analysis were employed. Two bands of 55 kDa and 45 kDa were detected in rat brain (Fig. 3C–D, lane 1); similar bands were also observed in bony fish brain extracts (Fig. 3C, lane 3). As has been previously demonstrated, the two GLUT1 isoforms represent the forms present in endothelial and brain cells [43]. In shark brain of *S. chilensis*, only one GLUT1 isoform of 50 kDa was detected in total brain extracts (Fig. 3C and lane 2) from the telencephalic cortex (Fig. 3D, lane 3), diencephalum (lane 4), mesencephalic tectum (lane 5), cerebellum (lane 6) and brain stem (lane 7). Thus, in shark brain only one GLUT1 isoform is expressed, which may suggest a preferential GLUT1 expression in endothelial or glial cells.

To analyze GLUT1 distribution and localization in the brain of different species, immunoperoxidase analysis was initially undertaken (Fig. 3E–H). In rat and bony fish brain, GLUT1 was highly detected in the endothelial cells that form the BBB (Fig. 3E, F); however, in shark brain (*S. chilensis* and *S. canicula*), GLUT1 was mainly detected in perivascular structures (Fig. 3G–H).

To conduct a detailed analysis of the pericapillary localization of GLUT1 in *S. chilensis* shark brain, endothelial cells and neurons were identified with propidium iodide (Fig. 3I) and the radial glia processes were identified with an anti-GLUT1 antibody (Fig. 3J). GLUT1-positive perivascular structures formed a linear reaction in some vessels (Fig. 2K, arrows) or spherical structures in others (Fig. 3L and inset). Similar results were seen in other regions of the brains, such as the brain stem (Fig. 3M, arrow and inset). Furthermore, quantitative analysis of perivascular GLUT1 reactivity was conducted. In a total of four *S. chilensis* shark brains, 9098 blood vessels were quantified; 3548 vessels were observed in the telencephalic cortex, 1209 in the diencephalon, 1705 in the mesencephalon, and 2699 in the cerebellum. In each of the areas analyzed, approximately 80% of the blood vessels were positive for GLUT1 within perivascular regions (Fig. 3N).

### GLUT1 is localized in the vascular end-feet of glial cells but not in brain endothelial cells

**Figure 4:**
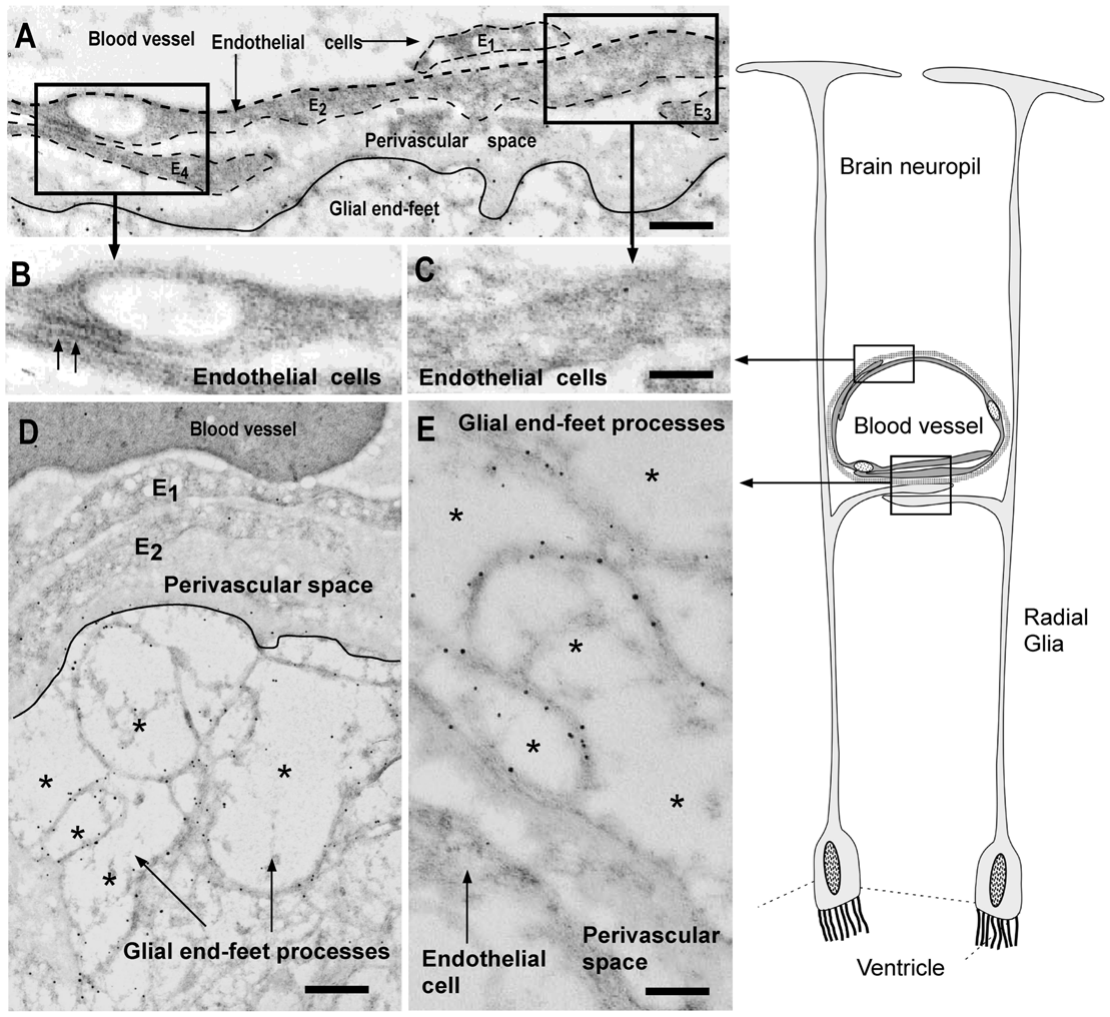
Ultrastructural immunocytochemistry of GLUT1 in shark brain. Immunohistochemical analysis using anti-GLUT1 antibody and anti-IgG labeled with 10-nm gold particles. A, Blood vessel of shark brain showing endothelial cells (E1–E4) and the perivascular space. B–C, High-power view of endothelial cells without immunoreaction. A junction complex is depicted (B, arrows). D–E, Perivascular space and glial end-feet processes (asterisks). The immunoreaction is mainly observed in the cellular membranes of the glial end-feet processes (asterisk). The schematic drawing shows radial glial cells and processes contacting the blood vessels. Scale bars: A–E, 1 µm.

Ultrastructural immunohistochemistry was used to define the localization of GLUT1 in endothelial and/or glial cells of *S. chilensis*. In blood vessels, the endothelial cells overlap, forming a continuous layer without tight junctions (Fig. 4A and B, arrows). GLUT1 was not observed in the endothelial cells of blood vessels by immunohistochemical analysis (Fig. 4B–C); however, it was observed in electron-lucent glial end-feet processes that contact perivascular blood vessels. The positive reaction for GLUT1 was concentrated in the cellular membrane, where gold particles of 10 nm in width were seen in the end-feet processes (Fig. 4D–E, asterisk).

### Apical and basolateral GLUT1 polarization in the choroid plexus cells at the CSF-brain-barrier

**Figure 5:**
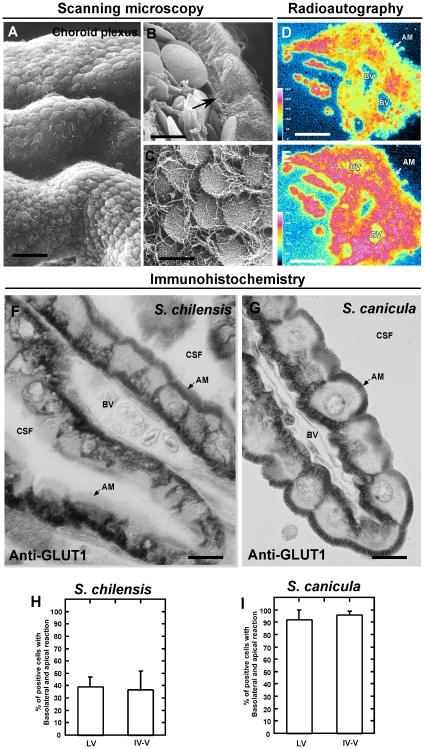
GLUT1 expression and function in the shark choroid plexus cells. A–C, Scanning electron microscopy of choroid plexus cells. The epithelial cells of the plexus show a close relationship with the blood vessel (B, arrow). Microvilli and cilia are observed in the apical membranes (C). D–E, Choroid plexus from lateral ventricle. Autoradiograph analysis 1 h after intravenous injection using ^3^H-2-deoxy-D-glucose (500 µCi)(D). The images show pseudocolor representation of consecutive sections (blue, negative signal; yellow, low signal; and red, high signal) after 1 (D) or 8 days (E) at 4°C (E) of consecutive 1 µ thick sections. F–G, Immunohistochemistry of GLUT1. GLUT1 is localized in the apical and/or basolateral membrane of choroid plexus epithelial cells. H–I, Quantitative analysis of choroid plexus cells with apical and basolateral GLUT1 polarization in *S. chilensis* and *S. canicula*. Data represent the means ± SD of % of GLUT1 positive cells in apical and basolateral membranes of choroid plexus cells (lateral and fourth ventricles) from four independent experiments. AM: apical membrane, BV: blood vessel, CSF: cerebrospinal fluid. Scale bars: A, 50 µm, B–C, F–G, 10 µm; D–E, 80 µm.

**Figure 6:**
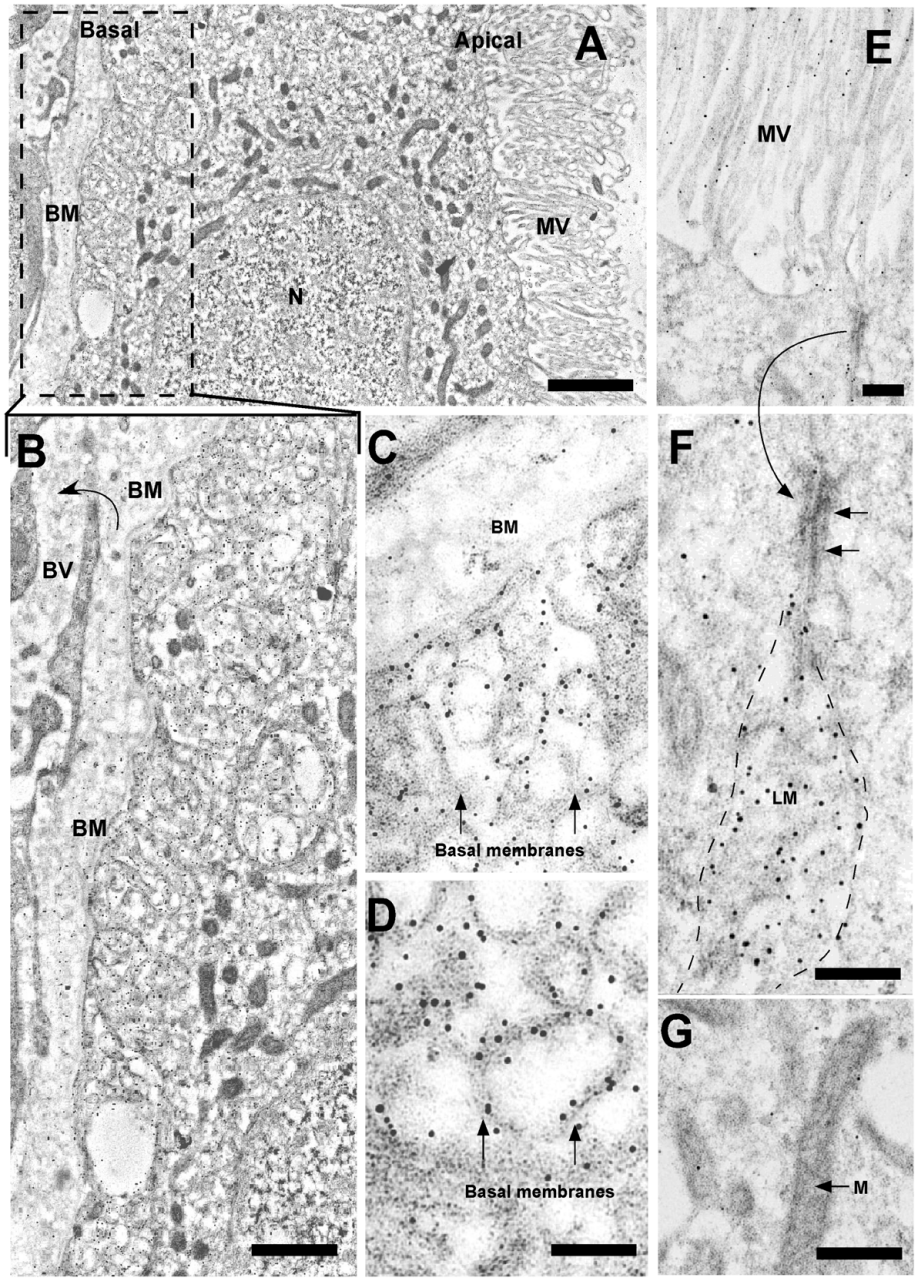
Ultrastructural immunohistochemistry of GLUT1 in shark brain choroid plexus cells. Immunohistochemical analysis using anti-GLUT1 antibody and anti-IgG labeled with 10-nm gold particles. A–B, Low magnification analysis of the epithelial cells and blood vessel. C–D, Basal region of the cell showing immunoreaction mainly in the cellular membranes (arrows). E, Apical region of the cells and microvilli. A junction complex is depicted (arrows) and observed with high magnification in F. G, Cytoplasm of the cell and mitochondria. The positive reaction was not detected in these structures. BM: basal membrane, BV: blood vessel, LM: lateral membrane, M: mitochondria, MV: microvilli, N: nucleus. Scale bars: A, 1 µm, B, 3 µm; C–G, 5 µm.

Scanning electron microscopy of *S. chilensis* demonstrated that the shark has a highly developed choroid plexus structure, with folds throughout the cerebral ventricles (Fig. 5A). The apical membrane of the choroid plexus cells contains cilia and small microvilli (Fig. 5A–C). In addition, blood vessels were observed in the proximity of the basolateral membranes of epithelial cells (Fig. 5B, arrow). To analyze if choroid plexus cells are actively involved in glucose uptake, their uptake of ^3^H-2-deoxy-D-glucose was analyzed. The positive signal observed after pseudocolor analysis represents the specific label after 1 or 8 days (Fig. 5D, E) emulsion exposure of 1 µm thick consecutive sections. As shown in Fig. 5E, the radioactively-labeled glucose was concentrated (red signal) in the choroid plexus cells, demonstrating that glucose is actively incorporated in the epithelial cells. The high glucose uptake is suggestive of increased GLUT expression in shark choroid. In multiple species, GLUT1 was primarily located at the basolateral membrane of choroid plexus cells. Furthermore, functional studies have suggested apical polarization; however, it has not been detected in mammals or other species. In the present study, immunoperoxidase analysis was used to analyze GLUT1 distribution and localization in the choroid plexus of *S. chilensis*. GLUT1 was primarily concentrated in the basolateral membrane (Fig. 5F); however, the transporter was also widely located in the apical membrane (Fig. 5F–G). Further quantitative analysis of the apical and basolateral distribution of GLUT1 in the brain of two species of sharks was undertaken. In a total of four *S. chilensis* shark brains, approximately 60% of the cells showed only basolateral polarization; however, 40% of the cells that form the choroid plexus in the lateral and fourth ventricle showed basolateral and apical polarization of GLUT1 (Fig. 5H). The bipolar localization of GLUT1 was even further evident in *S. canicula* shark brain; 90% showed GLUT1 basolateral and apical polarization in the choroid plexus cells of both ventricles (Fig. 5I). Finally, ultrastructural immunohistochemistry was used to define the specific localization of GLUT1 in the choroid plexus cells (Fig. 6). In the basal area, the cells have labyrinth-adapted cellular membranes, which concentrate the localization of GLUT1 (Fig. 6A–D, arrows). The undulating lateral membrane and microvilli also showed a positive reaction for GLUT1 (Fig. 5E and F), which was almost absent inside the epithelial cells (Fig. 6G).

### MCT1 expression in shark brain

Due to the GLUT1 expression pattern in *S. chilensis* brain, we postulated that this organ might generate lactate within glial cells to then be transferred to neuronal cells [9], [10], [35]. The glucose may enter into the glia across the BBB and may be actively transported from the CSF. In both cases, glucose preferentially enters glial cells and not neurons. Thus, shark brain may express functional MCTs to transfer these molecules from glia cells to neurons.

**Figure 7:**
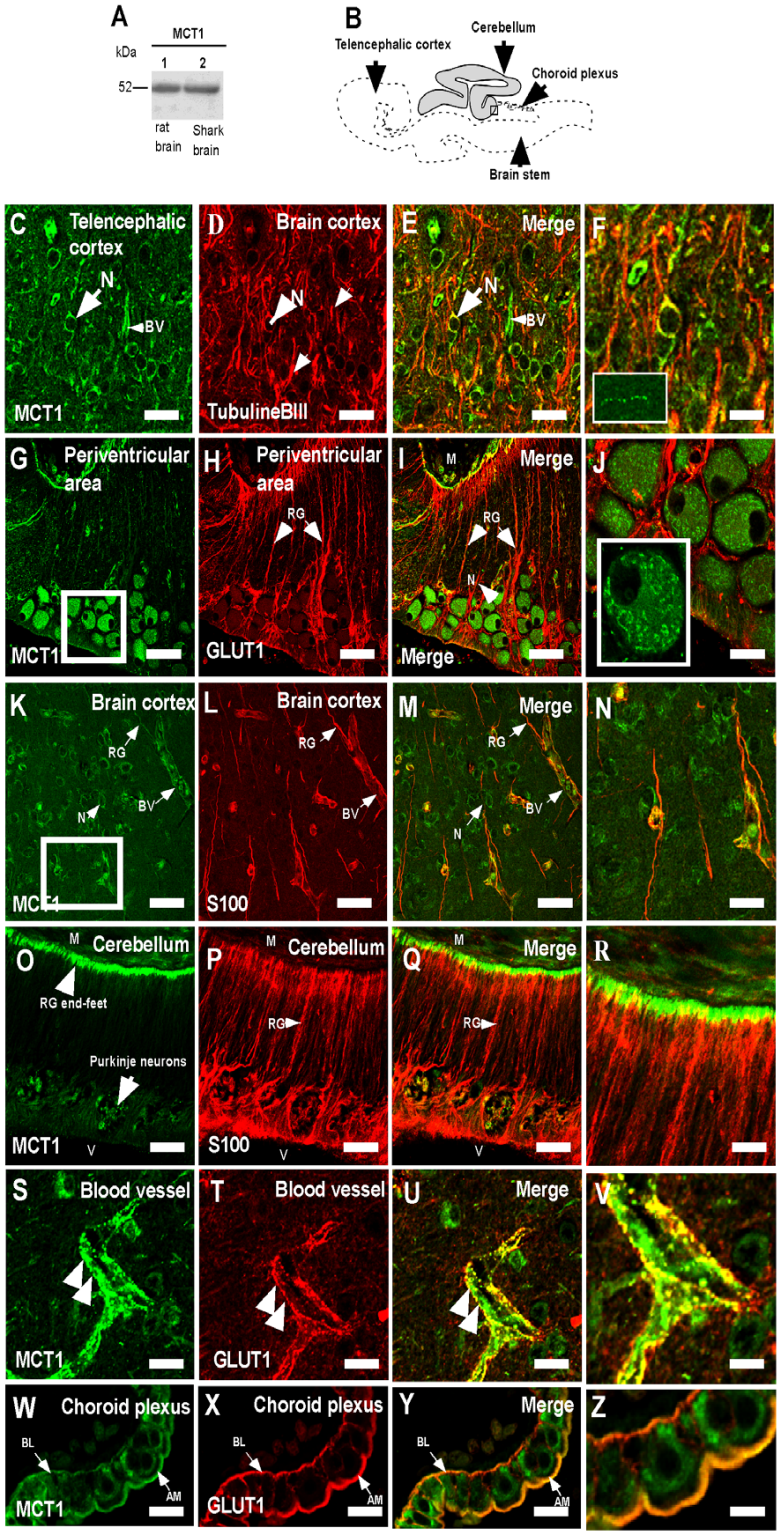
MCT1 is expressed in neurons, radial glia and endothelial cells. A, Western blot analysis of MCT1 expression from total protein extracts prepared from rat brain (lane 1) and shark brain (lane 2). B, Schematic representation of shark brain *S. chilensis*. C–Z, Immunohistochemistry and confocal microscopy analysis. MCT1 is observed in neurons of brain cortex (C–F), neuron of periventricular area (G–J), radial glia cells (K–N), glial end-feet of cerebellum (O–R), endothelial cells (S–V) and choroid plexus cells (W–Z). In glial end-feet (S–V, arrows) and choroid plexus (W–Z), MCT1 co-localized with GLUT1. BV: blood vessel, AM: apical membrane, BL, basolateral membrane M: meninges, N: neurons, RG: radial glia, V: ventricle. Scale bar: A–Q, 20 µm.

MCT1 expression in shark brain (*S. chilensis*) cellular extract was assessed by Western blot analysis; one band was identified at 52 kDa (Fig. 7A, lane 2), which was similar to that detected in rat brain (Fig. 7A, lane 1). MCT1 was amply localized in the brain within radial glia, choroid plexus, blood vessels and neurons. In neurons, MCT1 was localized in the cytoplasm with tubulin βIII in the soma (Fig. 7, C–F). Decreased MCT1 expression was also observed in dendritic projections (tubulin βIII-positive) (Fig. 7E) and axons (Fig. 7F, inset). Thus, MCT1 might be a transporter expressed in neurons that is preferentially expressed intracellularly. Indeed, in the large neurons present in the periventricular regions (mesencephalic roof) of the shark brain, MCT1 was localized intracellularly in structures that were similar to mitochondria and/or cytosolic storage vesicle (Fig. 7G–J, inset). In the radial glia from the *S. chilensis* brain, MCT1 was observed in their extensions, which co-localized with the S100 protein (Fig. 7K–N). In the cerebellum, the MCT1 signal was detected in the end-feet of the radial glia (Fig. 7O); a partial colocalization with S100 was detected (Fig. 7O–R).

The distribution of MCT1 at the vascular level was next assessed. MCT1 was detected in the endothelial cells and radial glial cell end-feet, which were also GLUT1-positive (Fig. 7S–V, arrows). Similar results were observed in the major and minor vessels in all of the brain. In the choroid plexus cells, MCT1 was localized in the apical and basolateral membrane of the epithelial cells (Fig. 7W–Z). In both membranes, MCT1 co-localized with GLUT1 (Fig. 7Y–Z). Thus, MCT1 is amply expressed in the shark brain.

### Differential expression and localization of MCT2 and MCT4 in shark brain

**Figure 8:**
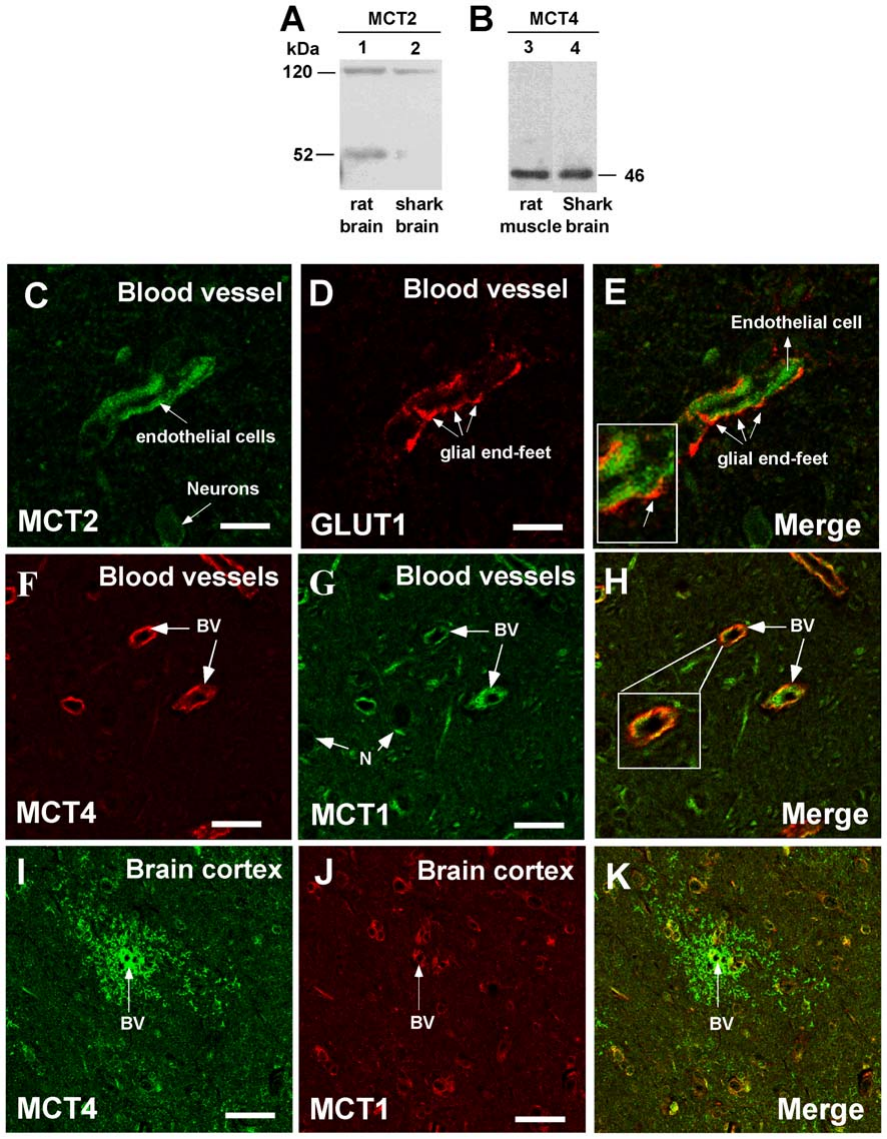
MCT2 and MCT4 are also expressed in shark brain cells. A–B, Western blot analysis of MCT2 and MCT4 expression in total protein extracts prepared from rat brain (A, lane 1), *S. chilensis* shark brain (A, B; lanes 2 and 4) and rat muscle (B, lane 3). C–K, Immunohistochemistry of MCT1, 2, 4, and GLUT1 in the telencephalic area. MCT2 is observed in endothelial cells and neurons (C) without co-localization with GLUT1 (C–E). MCT4 is observed in endothelial cells (F) and perivascular structures in telencephalic cortex vessels (I). MCT4 co-localized with MCT1 (H, K). BV: blood vessel, N: neurons. Scale bar: C–H, 15 µm; I–K, 50 µm.

**Figure 9:**
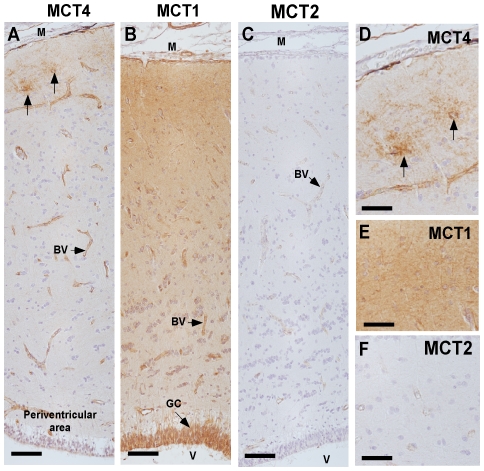
Comparative expression analysis of MCT1, 2 and 4. A, MCT4 detection in brain cortex of *S. chilensis* by immunohistochemistry. The reaction is observed in blood vessels and rosette-like structures (arrows). B, MCT1 detection in the brain cortex of *S. chilensis*. The reaction is detected in blood vessels and glial cells. C, MCT2 analysis in the brain cortex of *S. chilensis*. The positive reaction is weakly detected in blood vessels. D–F, High-power view images of telecephalic area and meningeal surface. BV: Blood vessel. GC: glial cells. M: meningeal surface. V: lateral ventricle. Scale bar: A–C, 100 µm; D–F, 30 µm.

MCT2 was weakly detected in *S. chilensis* shark brain (Fig. 8 and 9). In rat brain, the transporter had a molecular weight of 52 kDa (Fig. 8A, lane 1), which was almost undetectable in shark brain (Fig. 8A, lane 2). Additionally, a protein of 120 kDa was detected, similar to previously reports in rat brain (Fig. 8A, lane 1). MCT2 was weakly detected in the telencephalic cortex and the brain stem (Fig. 9), while it was clearly expressed by endothelial cells and neurons (Fig. 8C). Immunohistochemical analysis showed low MCT2 and GLUT1 co-localization (Fig. 8C–E and inset) in the glial end-feet-endothelial cell junction alone.

**Figure 10:**
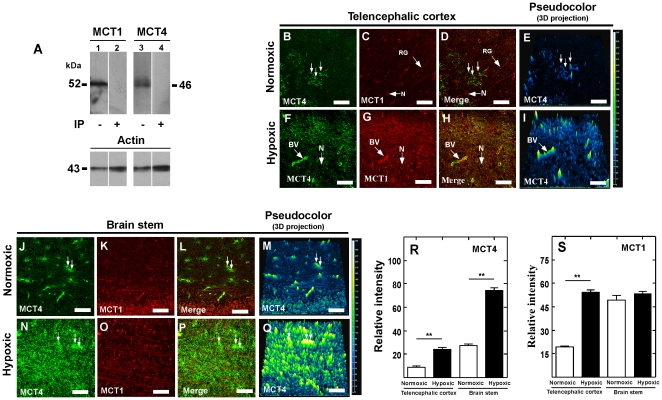
Hypoxia increases MCT1 and MCT4 in shark brain. A, Western blot analysis of MCT1 and MCT4 expression in total protein extracts prepared from shark brain. MCT1 (lane 1), MCT4 (lane 3). Negative controls were performed with primary antibodies preabsorbed with inductor peptides (lanes 2 and 4). B–Q, Immunofluorescence and confocal analysis of MCT1 and MCT4 in telencephalic cortex (B–I) and brain stem (J–Q) in normoxic or hypoxic conditions. Pseudocolor analysis and three-dimensional projection images are also included (blue color, low reaction; green-yellow color, high reaction; E, I and M, Q). R, S, Quantitative analysis of the MCT4 and MCT1 immunoreaction in normoxia and hypoxia. **p<0.001, one tailed t-test. Data represent the means ± SD from four independent determinations. BV: blood vessel, GR: radial glia, N, neuron. Scale bar: A–Q, 50 µm.

Western blot analysis revealed MCT4 expression with a molecular weight of 46 kDa, which was the same size observed in rat muscle (Fig. 8B, lanes 3 and 4). MCT4 was highly expressed in endothelial cells (Fig. 8F–H and 9), and a clear co-localization of MCT1 and MCT4 was observed (Fig. 8F–H and inset). In the external region of the telencephalic cortex of *S.chilensis* shark, MCT4 was mainly observed in “rosette-like” structures, surrounding the blood vessels (Figs. 8I–K and 9A, arrows). These structures were not observed with anti-MCT1 or anti-MCT2 antibodies (Fig. 9, D–F, arrows). Similar structures were also observed in the telencephalic cortex and brain stem of *S. canicula* (Fig. 10). We hypothesized that these “rosette-like” structures represent a high concentration of glial end-feet surrounding the blood vessels, which were MCT1-negative.

### Modified MCT1 and MCT4 expression and distribution in shark brain under hypoxic conditions

Under hypoxic conditions, no changes in GLUT1 immunohistochemical staining in the shark brain were observed (data not shown). Quantification of the apical and basolateral distribution of GLUT1 in 2078 (n = 3) choroid plexus cells of the lateral ventricle and in 1803 (n = 3) choroid plexus cells of the fourth ventricle revealed that over 90% of the cells maintained GLUT1 bi-polarization and the same intensity in the immunoreaction.

The effects of hypoxia on MCT1 and MCT4 expression were analyzed in *S. canicula* shark brain, detecting proteins with molecular weights of 52 and 46 kDa, respectively (Fig. 10A). In normoxic animals, MCT4 was detected in telencephalic rosette-like structures (Fig. 10B–E, arrows) and in the brain stem perivascular regions (Fig. 10J–M, arrows). These structures were MCT1-negative (Fig. 10C and K). Increased MCT4 immunostaining in the telencephalic cortex and the brain stem was observed in hypoxic animals (Fig. 10F and N). Higher MCT1 immunoreaction in the brain cortex was also observed along with a clear MCT1 and MCT4 co-localization (Fig. 9G–H). No changes were observed in the brain stem (Fig. 10P–Q).

The effect produced by hypoxia was measured semi-quantitatively by applying the pseudocolor function of the Nis-Element software and analyzing the image in three-dimensional projection (Fig. 10E and I; M and Q). In hypoxic animals, MCT4 was increased 3fold in the telencephalic cortex (intensity value, 24.4±1.2 and control 8.7±0.3, p<0.01, n = 3) and brain stem (intensity value, 74.2±2.6 and control 27.2±1.4, p<0.01, n = 3) (Fig. 9R). Similarly, MCT1 increased 4-fold in the telencephalic cortex (Fig. 10S); however, no changes were detected in the brain stem (Fig. 10S).

**Figure 11:**
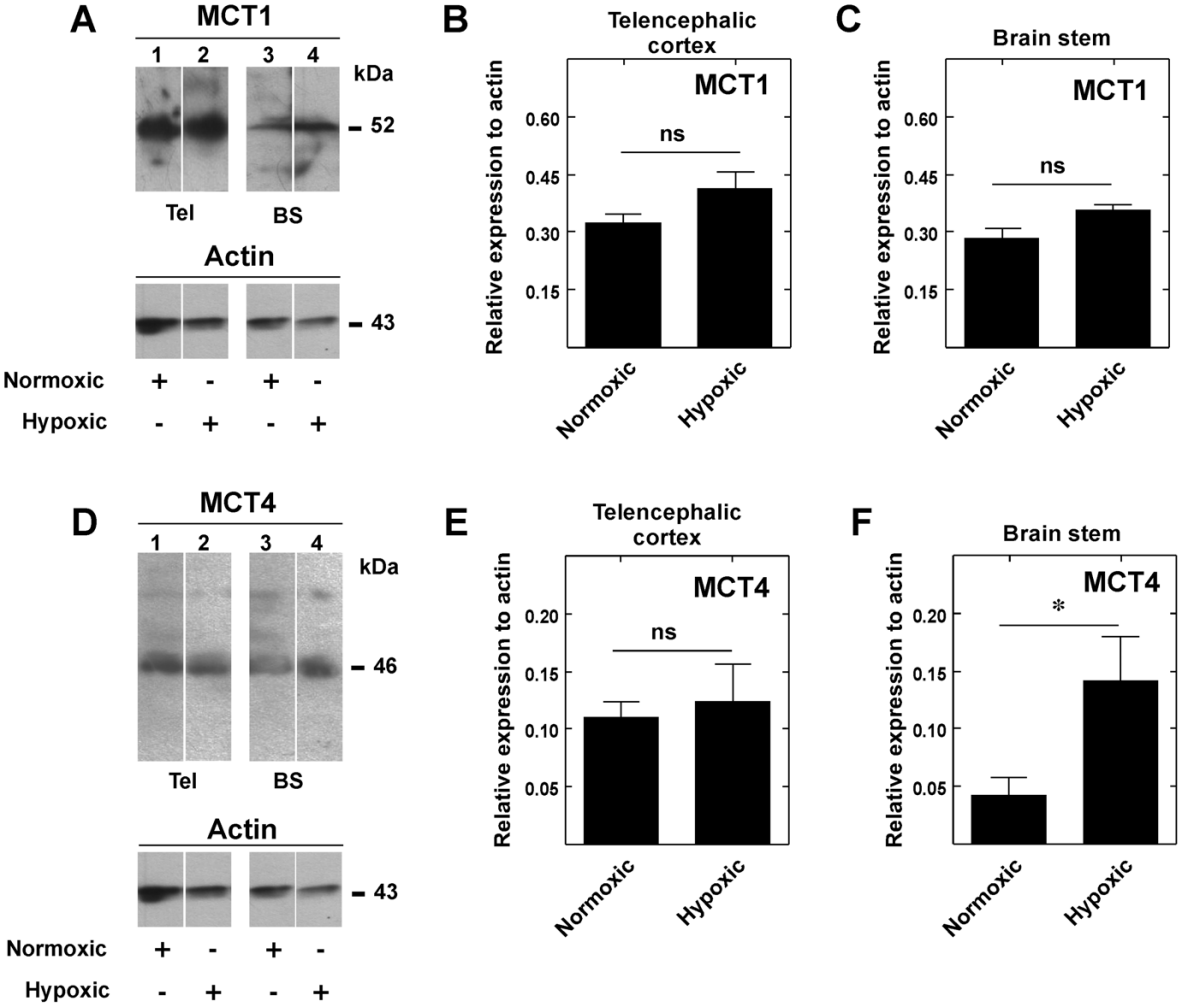
Hypoxia changes MCT1 distribution in brain cortex and increases MCT4 expression in brain stem. A and D, Western blot analysis of MCT1 and MCT4 expression in total protein extracts from telencephalic cortex (Tel, lanes 1, 2) and brain stem (BS, lanes 3, 4) in normoxic and hypoxic conditions. B–C and E–F. Quantitative analysis of the MCT1 and MCT4 reaction in normoxic and hypoxic condition. The hypoxic condition increased MCT4 expression in the brain stem. *p<0.05, one tailed t-test. Data represent the means ± SD from three independent determinations.

To determine if MCT1 and MCT4 immunoreaction changes in response to hypoxia were produced by a differential distribution of the transporters in the brain tissue or induced expression, Western blot analysis using protein extracts isolated from telencephalic cortex or brain stem of normoxic or hypoxic *S. canicula* was performed. In hypoxic animals, changes in MCT1 expression in telencephalic cortex (Fig. 11A, lanes 1, 2 and B) or brainstem (Fig. 11A, lanes 3, 4 and C) were not observed, suggesting that hypoxia influences MCT1 distribution rather than expression level. Changes in MCT4 expression in the telencephalic cortex were not detected (Fig. 11D, lanes 1, 2 and E); however, MCT4 expression increased 3-fold in the brain stem of hypoxic animals (Fig. 11D, lanes 3, 4 and F).

## Discussion

In this study, the expression and distribution of the glucose transporter, GLUT1, at both the optical and ultrastructural levels was characterized in the radial glial cells that make up the BBB and the epithelial cells that form the blood-CSF barrier in shark brain. Glucose uptake was also analyzed using radioactive glucose and autoradiography. Finally, the localization of MCT1, MCT2, and MCT4 was assessed in shark brain and the changes produced under hypoxic conditions were defined.

**Figure 12:**
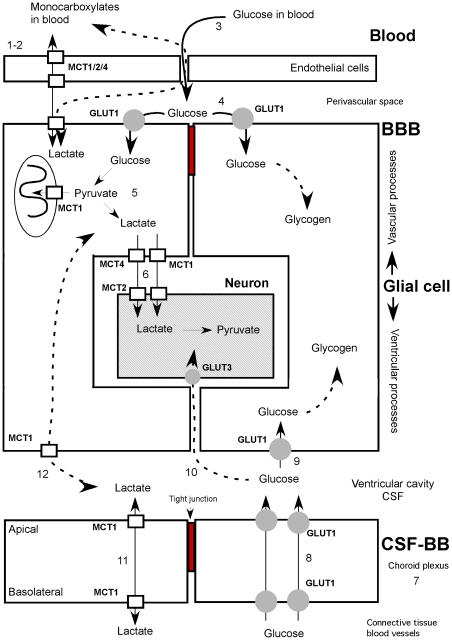
Model for glucose and monocarboxylate movement within shark brain. **At the level of the BBB:** 1. The endothelial cells of shark brain do not express GLUT1 as do those of bony fishes and mammals. 2. The endothelial cells of shark brain express MCT1, 2 and 4, thereby incorporating monocarboxylates from the blood. 3. The endothelial cells do not have tight junctions; the glucose diffuses to the pericapillary space. 4. The glucose of the blood enters the radial glial cells through the GLUT1, a polarized transporter in the perivascular processes. 5. Within the radial glia, the glucose is metabolized to pyruvate and/or lactate. 6. Lactate could exit the radial glia using MCT4/MCT1 and be incorporated by the neurons using MCT2. MCT1 is responsible for the entrance of both substrates to the interior of the mitochondria. Dotted lines, alternative mechanisms. **At the level of the blood-CSF barrier:** 7. Shark brain is able to capture glucose from the blood through the choroid plexus (blood-CSF barrier). 8. The glucose is transferred vectorially to the CSF due to the basolateral and apical polarization of GLUT1. 9. The glucose present in the CSF enters the glia through GLUT1 localized in the periventricular bodies of the radial glia. 10. The glucose could diffuse to the extracellular space through the ventricular wall and reach neurons present in the brain tissue. 11. The choroid plexus shows MCT1 at the basolateral and apical levels, allowing entrance and exit flows of monocarboxylates, depending on the relative concentrations of these compounds. 12. MCT1 is localized at the ventricular level in the radial glia, allowing the capture or efflux of lactate from the CSF.

Early studies of shark brain determined that changes in vascular glucose concentrations generated higher glucose concentrations in the CSF [44]. The choroid plexus, which constitutes 2.4% of the brain weight (a higher percentage than is described for mammals), may play a very important role in the incorporation of glucose to shark brain [5], [45], [46]. Autoradiography analysis indicated that the epithelial cells of the plexus are capable of incorporating ^3^H-2-deoxy-D-glucose, confirming previous observations [44]. At both the optical and ultrastructural levels, immunohistochemical analyses demonstrated that shark choroid plexus presents a labyrinth of basolateral membranes containing the glucose transporter, GLUT1 [19], [20], [22] (Fig. 12). Surprisingly, GLUT1 was also detected in the apical membrane of choroid plexus epithelial cells, which represents the first evidence of GLUT1 at this cellular region. This apical localization of GLUT1 allows a rapid vectorial flow of glucose between the basolateral and apical membranes, transporting the glucose from the blood to the CSF. It also indicates that the transfer of glucose to the CSF may be through a finely regulated mechanism relying on the differential *sorting* of GLUT1 to the apical membrane. Once the glucose has entered the CSF, it is captured by the periventricular glial cells, which also express GLUT1. This represents an efficient mechanism of glucose entry into shark cerebral parenchyma.

Radial glial cells and astrocytes are found in the brain of all vertebrate groups [47], [48]. During pre-natal development, radial glial cells function like stem cells of the CNS and also provide metabolic support to differentiating neurons [49], [50], [51]. In the post-natal brain, astrocytes replace the radial glia. However, in some species (e.g., sharks and several lizards), these cells remain in the adult brain [52], [53]. The radial glia of the shark brain has been identified using different markers like S100, GFAP and GS [54], [55]

The BBB is formed by the endothelium in all vertebrates except sharks and rays, in which it is formed at the glial level [1], [2], [3], [5] (Fig. 12). The BBB of the shark, *Squalus acanthias*, is known to transport 3-O-methyl-D-glucose in a saturable, stereospecific manner [6]. These data allow us to propose that a GLUT-type transporter mediates the transfer of glucose to shark brain. GLUT1 is highly expressed in the endothelial cells of the cerebral capillaries of mammals, reptiles, and bony fishes [19], [56], [57], [58], [59]. Nonetheless, the present study shows that in sharks, GLUT1 is expressed in the perivascular region, specifically in the processes of the radial glial cells that contact the blood capillaries (Fig. 12), as observed through fluorescence microscopy and ultrastructural immunocytochemistry. Because shark glial processes possess tight junctions, glucose enters the radial glia directly [60], [61] and not the cerebral parenchyma as occurs in mammals and bony fishes. Although little is known about many aspects of glucose metabolism in radial glial cells, it is possible that these cells store glycogen, a molecule that is metabolized upon neuronal activation [62], [63]. Furthermore, the expression of enzymes related to the glycogen metabolism has been found in the brain of rays [47], [64], [65].

The central question about the metabolism within the shark brain is how glucose or some of its metabolites reach the neuron. We propose that the shark brain develops a metabolic glia-neuron coupling; this model involves the incorporation of glucose into the glial cells and the transfer of metabolic derivatives, such as lactate or ketone bodies, to the neuron. Because this model has been postulated for mammalian brain [8], [9], [61], [66], attempts to test this hypothesis require analysis of MCT (responsible for the influx and/or efflux of lactate) distribution in shark brain.

Studies of shark brain suggest that it metabolizes ketone bodies [64]. Prior to the present study, MCT expression in the brain had not been analyzed in non-mammalian vertebrates. Here, Western blot analysis using antibodies specific for MCT1, MCT2, and MCT4 detected proteins similar to those observed in the positive controls. In addition, the presence of MCT1 in the apical and basolateral membranes of the choroid plexus was observed (Fig. 12), indicating that MCT1 may be involved in the movement of ketone bodies at the blood-CSF barrier. If lactate was generated by radial glial cells, the gradient of this metabolite would be oriented from the CSF to the blood such that the plexus would be involved in the depuration of the excess lactate generated by the cerebral parenchyma. MCT1 has also been described in mammalian endothelial cells involved in the transport of ketone bodies through the BBB [29], [30], [32]. In this study, the expression of MCT1 and MCT2 in the endothelial cells of shark brain was observed. Whereas MCT1 and MCT4 could be related to the influx or efflux of lactate [35], MCT2 could strengthen the influx of lactate, particularly given the low concentrations of ketone bodies at the vascular level [26], [27], [67]. Because the BBB is not present within endothelial cells, we propose that these cells have high- and low-affinity MCTs involved in the influx of monocarboxylates to the vascular endothelium and not to the cerebral parenchyma (Fig. 12).

The localization of MCT1 and MCT4 in the astrocytes of mammalian brain has been described [7], [26], [35], [68]. Likewise, expression of these transporters was observed in shark radial glial cells, which was associated mainly with the glial processes that contact the blood vessels. MCT1 is involved in the influx or efflux of ketone bodies by glial cells, a function that was previously reported for astrocytes [7], [30], [69]. Furthermore, immunofluorescence analysis showed that MCT1 is localized in glial processes that contact the blood vessels or in perivascular processes that form *rosette*-type structures [70]. Thus, it is feasible to propose that radial glial cells deliver ketone bodies or lactate to the cerebral parenchyma considering the involvement of MCT4 in the efflux of lactate [27], [35], [71], [72] (Fig. 12).

The metabolic characteristics of shark radial glia have not been described to date. However, efflux of lactate from the brain has been reported under starvation conditions [64], suggesting that radial glia may generate important concentrations of lactate that can reach the neuron or be eliminated by the blood [64]. MCT2 is a high-affinity transporter involved in the influx of lactate through the cellular membrane whose expression has been described in mammalian neurons [36], [37]. Moreover, the expression of MCT1 has been shown in the hypothalamic nucleus [33], [35]. Both transporters are expressed in shark neurons. Specifically, the reactivity pattern of MCT1 suggests strongly that it is located in the mitochondria, as has been shown in skeletal muscle and in neurons of the thalamus of rats [73], [74]. MCT2, in turn, is located in the plasma membrane, which permits the incorporation of lactate or ketone bodies (from blood) to the neurons.

Different data indicate an increased MCTs expression, mainly MCT4, in hypoxic conditions of mammals and fishes tissues [38], [39], [40]. In mammals, the MCT4 gene promoter contains elements responsive to hypoxia, which is similar to that described for HIF-1α [39], [42]. Similar results have been showed for GLUT1 in mammals [75], [76], however, there are not information in shark brain about MCTs and GLUTs expression in hypoxic condition. In our study, different variations were found for MCT1 and MCT4 in response to hypoxia, however, we were not able to observe GLUT1 modification. Although we did not study expression levels of mRNA for MCT1 in response to hypoxia, Western blot and immunohistochemical analysis led us to suggest that MCT1 protein levels do not change, only their distribution is modified in the telencephalon. These results suggest that MCT1 may modulate its cellular localization dynamically, adapting lactate influx and/or efflux of shark brain cells under anaerobic metabolism. Recently, it has been postulated that MCT1 increases mRNA expression in cultured rat astrocytes during hypoxia [77], and also in hypoxic training [78], however in zebrafish brain it has not been observed (Ngan et al. 2009). These results indicate that fishes and mammals may regulate differentially the expression and distribution of MCT1 under hypoxia in brain cells.

In mammals, MCT4 is a low-affinity transporter involved mainly in lactate efflux from astrocytes. In the gills of the teleost fish, *Danio rerio* (zebrafish) [38], changes in MCT4 mRNA expression levels have been reported in response to hypoxia. Similar results were observed in our study, specifically in brain stem of shark after hypoxia. These results and the presence of the five LDH isoenzymes in elasmobranchs [79] is suggestive that lactate production could increase under hypoxia and the cells use MCT4 to remove lactate from the glia.

In conclusion, our data allow us to propose a novel coupled metabolic model in shark brain. The distribution of GLUT1 and MCT1, 2 and 4 in the shark brain strongly support that glial cells may incorporate glucose and release lactate to be used by the neuron. This metabolic coupling mechanism would be an efficient way to feed a brain that has the BBB in the glial cells.

## Materials and Methods

### Animals

In this study, all animals were handled in strict accordance with the Animal Welfare Assurance and all animal work was approved by the appropriate Ethics and Animal Care and Use Committee of the University of Concepcion, Chile (permit number 2010101A).

Male adult Sprague-Dawley rats were used for the experiments. Animals were kept in a 12-h light/dark cycle with food and water *ad libitum*. Twenty specimens of *Schroedericthys chilensis* (Pacific sea shark) were captured in the harbor of Concepción, Chile. Thirty two specimens of *Scyliorhinus canicula* (Mediterranean sea shark) were captured in the harbor of Malaga, Spain. Three specimens of *Oncorhynchus mykiss* (salmon) were donated from the Camanchaca Company, Tome, Chile. The animals were anesthetized with Ethyl-p-aminobenzoate (Sigma-Aldrich, St. Louis, MO, USA) dissolved in seawater. To induce hypoxia, ten *S. canicula* sharks were kept in seawater after capture from which the oxygen was eliminated [80].

### Immunocytochemistry

The brain of different species were fixed in Bouin solution (750 mL of saturated picric acid, 250 mL of formaldehyde 37%, and 50 mL of glacial acetic acid), and the samples were dissected and post-fixed by immersion for 12 h. After post-fixation, the samples were dehydrated in graded alcohol solutions and embedded in paraffin. The sections (7 µm) were obtained and mounted on poly-L-lysine-coated glass slides. Before immunostaining, the sections undergoing peroxidase immunohistochemistry were treated with 3% hydrogen peroxide in absolute methanol to inactivate endogenous peroxidase activity.

For immunohistochemical analyses, the following antibodies and dilutions were used: rabbit anti-GLUT1 (1∶100, Alpha Diagnostic International, INC., San Antonio, TX, USA), rabbit anti-S100a (1∶500, DAKO, Campintene, CA, USA), rabbit anti-glial fibrillary acidic protein (GFAP; 1∶200, DAKO), mouse anti-vimentin (1∶200, DAKO), mouse 3CB2 (1∶100, this antibody was developed by Francisco A. Prada, and was obtained from the Developmental Studies Hybridoma Bank maintained by the Department of Biological Sciences, University of Iowa, Iowa City, IA, USA), chicken anti-MCT1 (1∶100, Millipore, Temecula, CA, USA), chicken anti-MCT2 (1∶50, Millipore), and rabbit anti-MCT4 (1∶20, Millipore). The antibodies were diluted in a Tris-HCl buffer (pH 7.8) containing 8.4 mM sodium phosphate, 3.5 mM potassium phosphate, 120 mM sodium chloride, and 1% bovine serum albumin (BSA). Sections were incubated with the antibodies overnight at room temperature in a humid chamber. After extensive washing, the sections were incubated for 2 h at room temperature with the appropriate peroxidase-labeled secondary antibody (1∶500; Jackson ImmunoResearch Laboratories, INC., Pennsylvania, USA). The peroxidase activity was developed using a DAB substrate kit (ImmunoPure; PIERCE Biotechnology, Rockford, IL, USA). For immunofluorescence and colocalization analyses, the tissues were incubated with the primary antibodies overnight and subsequently with Cy2-, Cy3- or Cy5-labeled secondary antibodies (1∶200; Jackson ImmunoResearch Laboratories). Some samples were counter-stained with propidium iodide (1∶1000). The slides were analyzed using confocal laser microscopy (D-Eclipse C1 Nikon, Tokyo, Japan).

### Reverse transcription-polymerase chain reaction

Total RNA from the telencephalic cortex and control tissues (rat brain), were isolated using Trizol (Invitrogen, Rockville, MD, USA). For RT-PCR, 2 µg RNA was incubated in a 20 µL reaction volume containing 5× buffer for M-MulV reverse transcriptase, 20 U RNAse inhibitor, 1 mM dNTPs, 2.5 µM oligo(dt)18 primer, and 10 U revertAidTM H minus M-MuLV reverse transcriptase (Fermentas International INC., Burlington, Ontario, Canada) for 5 min at 37°C followed by 60 min at 42°C and 10 min at 70°C. Parallel reactions were performed in the absence of reverse transcriptase to control for the presence of contaminant DNA. For amplification, 1αL cDNA aliquot in a total volume of 12.5 µL containing 10× PCR buffer without MgCl_2_, 10 µM dNTPs, 25 µM MgCl_2_, 0.3125 U Taq DNA pol (Fermentas International), and 10 µM of each primer was incubated at 95°C for 5 min followed by 35 cycles of 30 s at 95°C, 30 s at 55°C, and 30 s at 72°C and a final extension of 7 min at 72°C. PCR products were separated by 1.2% agarose gel electrophoresis and visualized by staining with ethidium bromide. The following sets of primers were used: GLUT1a, sense 5′- GTGTCATCAATGCCCCACAG -3′ and antisense 5′- GCCAATAATAAACCGTCCCA - 3′ (expected product of 281 bp); GLUT1b, sense 5′- GCGGAATTCAATGCTGATGAT -3′ and antisense 5′- AGGGCAGAAGGGCAACAGGAT -3′ (expected product of 350 bp).

### Immunoblotting

Total protein extracts were obtained from rat brain, different regions of shark brain, as well as salmon brains. Tissues were homogenized in buffer A (0.3 mM sucrose, 3 mM DTT, 1 mM EDTA, 100 µg/mL PMSF, 2 µg/mL pepstatin A, 2 µg/mL leucopeptin, and 2 µg/mL aprotinin), sonicated three times on ice at 300 W (Sonics & Material INC, VCF1, Connecticut, USA) for 10 s, and separated by centrifugation at 4000×g for 10 min. Supernatant was centrifuged at 180,000× g for 45 min at 4°C. Proteins were resolved by SDS-PAGE (50 µg/lane) in a 5–15% (w/v) polyacrylamide gel, transferred to PVDF membranes (0.45 µm pore, Amersham Pharmacia Biotech., Piscataway, NJ, USA), and probed for 8 h at 4°C with rabbit anti-GLUT1 (1∶500), chicken anti-MCT1 (1∶1000), chicken anti-MCT2 (1∶500) or rabbit anti-MCT4 (1∶500) antibodies. After extensive washing, the PVDF membranes were incubated for 1 h at 4°C with peroxidase-labeled anti-chicken IgY (1∶1000; Jackson Immuno Research) or peroxidase-labeled anti-rabbit IgG (1∶5000; Jackson Immuno Research). The reaction was developed using the enhanced chemiluminescence (ECL) Western blot analysis system (Amersham Biosciences, Pittsburgh, PA, USA). Negative controls consisted of incubating the membrane with a pre-absorbed antibody (anti-GLUT1 1∶500, anti-MCT1 1∶100 or MCT4 1∶500) with 100 µg/mL inductor peptide (Millipore) incubated at 4°C overnight.

### Radioautography

Ten *S. chilensis* shark were anesthetized and injected with different activities (60–500 αCi) of [^3^H] 2-deoxy-D-glucose (specific activity 29.7 Ci/mmol) in the caudal peduncule. The label time was between 10–90 min. The brain was removed quickly and fixed in 4% paraformaldehyde and 2% glutaraldehyde in elasmobranch buffer [81] overnight at 4°C. After washing the choroid plexus in 0.1 M phosphate buffer, pH 7.4 containing 10% sucrose and 0.005% CaCl_2_, the tissues were incubated in 2% OsO_4_ for 2 h and then rinsed in 0.1 M phosphate buffer, pH 7.4. Following dehydration, the tissues were embedded in Epon-Araldite. Light microscope autoradiographs were prepared with serial semi-thin cuts (1αm) [82] coated with Hypercoat EM1 (Amersham) emulsion, according the manufacturer's instructions, and exposed for 1 days–4 weeks at 4°C. The films were developed with D19 developer (Kodak) for 7 min, stopped with 0.5% acetic acid, and fixed with fixer solution (Kodak). Some sections were stained with basic toluidine blue.

### Ultrastructural Immunohistochemistry

Brain tissues were immersed for 2 h in fixative containing 2% paraformaldehyde and 0.5% glutaraldehyde in elasmobranch buffer [81], containing 150 mM phosphate buffer, pH 7.4, 360 mM urea and 70 mM sodium chloride.

The samples were dehydrated in dimethylformamide and embedded in London Resin Gold (Electron Microscopy Science, Washington, DC). Ultrathin sections were mounted on uncoated nickel grids and processed for immunocytochemistry [83]. For immunostaining, the anti-GLUT1 antibody (1∶100) was diluted in incubation buffer consisting of Tris-HCl (pH 7.8) containing 8.4 mM sodium phosphate, 3.5 mM potassium phosphate, 120 mM NaCl and 1% BSA. After extensive washing, the ultrathin sections were incubated for 2 h at room temperature with 10-nm colloidal gold-labeled anti-rabbit IgG (1∶20). Uranyl acetate/lead citrate was used as contrast, and samples were analyzed using a Hitachi H-700 electron microscope (Hitachi, Tokyo, Japan) with 125–200-kV accelerating voltage. For scanning electron microscopy, the brain tissues were immersed for 2 h in fixative containing 4% paraformaldehyde and analyzed using a Etec Autoscan SEM (Etec Corp., Hayword, CA, USA).

### Generation of 3D ultrastructural data

Briefly, brain tissue from shark (*Schroedericthys chilensis*) was fixed by immersion using a mixture of 2% glutaraldehyde and 2% p-formaldehyde in elasmobranch buffer. After washing several times in PBS, 100 µm tissue sections were obtained using a Leica vibratome. These sections were then incubated with osmium (1%, 1 hr) to improve ultrastructure and contrast. After washing abundantly with distilled water, the brain sections were dehydrated with ascending concentrations of alcohol and incubated with propylene oxide to allow plastic infiltration (Epon). Once plasticized, the sections were cured at 60°C for 2–3 days. Ultrathin serial sections were cut at 49 nm and collected automatically using an automated tape collection ultramicrotome (ATUM, Hayworth et al., 2007). Ultrathin sections were then post-stained with lead citrate, carbon coated using Edward carbon evaporator, placed on a silicon wafer and inserted into an Electron Microscope (FE-SEM, Zeiss, Germany). Once the region of interest (ROI) was identified, it was imaged (beam parameters: 9 kV, 400 pA) automatically using backscattered electron detection. Serial images (6000×6000 pixels at 10 nm pixel size) were handled offline to generate a 3D image stack; affine transformation was used to align all serial images. Finally, the 3D image stack was segmented manually using Reconstruct version 1.1.0 [84].
